# Outcome Prediction Using Multi-Modal Information: Integrating Large Language Model-Extracted Clinical Information and Image Analysis

**DOI:** 10.3390/cancers16132402

**Published:** 2024-06-29

**Authors:** Di Sun, Lubomir Hadjiiski, John Gormley, Heang-Ping Chan, Elaine Caoili, Richard Cohan, Ajjai Alva, Grace Bruno, Rada Mihalcea, Chuan Zhou, Vikas Gulani

**Affiliations:** 1 Department of Radiology, University of Michigan, Ann Arbor, MI 48109, USA; lhadjisk@med.umich.edu (L.H.); jrgorm@umich.edu (J.G.); chanhp@med.umich.edu (H.-P.C.); caoili@med.umich.edu (E.C.); rcohan@med.umich.edu (R.C.); gmbruno@umich.edu (G.B.); chuan@med.umich.edu (C.Z.); vikasgulani@med.umich.edu (V.G.); 2 Department of Internal Medicine-Hematology/Oncology, University of Michigan, Ann Arbor, MI 48109, USA; ajjai@med.umich.edu; 3 Department of Electrical Engineering and Computer Science, University of Michigan, Ann Arbor, MI 48109, USA; mihalcea@umich.edu

**Keywords:** large language models, radiomics, deep learning, survival prediction, bladder cancer

## Abstract

**Simple Summary:**

Predicting the survival of bladder cancer patients following cystectomy can offer valuable information for treatment planning, decision-making, patient counseling, and resource allocation. Our aim was to develop large language model (LLM)-aided multi-modal predictive models, based on clinical information and CT images. These models achieved performances comparable to those of multi-modal predictive models that rely on manually extracted clinical information. This study demonstrates the potential of employing LLMs to process medical data, and of integrating LLM-processed data into modeling for prognosis.

**Abstract:**

Survival prediction post-cystectomy is essential for the follow-up care of bladder cancer patients. This study aimed to evaluate artificial intelligence (AI)-large language models (LLMs) for extracting clinical information and improving image analysis, with an initial application involving predicting five-year survival rates of patients after radical cystectomy for bladder cancer. Data were retrospectively collected from medical records and CT urograms (CTUs) of bladder cancer patients between 2001 and 2020. Of 781 patients, 163 underwent chemotherapy, had pre- and post-chemotherapy CTUs, underwent radical cystectomy, and had an available post-surgery five-year survival follow-up. Five AI-LLMs (Dolly-v2, Vicuna-13b, Llama-2.0-13b, GPT-3.5, and GPT-4.0) were used to extract clinical descriptors from each patient’s medical records. As a reference standard, clinical descriptors were also extracted manually. Radiomics and deep learning descriptors were extracted from CTU images. The developed multi-modal predictive model, CRD, was based on the clinical (C), radiomics (R), and deep learning (D) descriptors. The LLM retrieval accuracy was assessed. The performances of the survival predictive models were evaluated using AUC and Kaplan–Meier analysis. For the 163 patients (mean age 64 ± 9 years; M:F 131:32), the LLMs achieved extraction accuracies of 74%~87% (Dolly), 76%~83% (Vicuna), 82%~93% (Llama), 85%~91% (GPT-3.5), and 94%~97% (GPT-4.0). For a test dataset of 64 patients, the CRD model achieved AUCs of 0.89 ± 0.04 (manually extracted information), 0.87 ± 0.05 (Dolly), 0.83 ± 0.06~0.84 ± 0.05 (Vicuna), 0.81 ± 0.06~0.86 ± 0.05 (Llama), 0.85 ± 0.05~0.88 ± 0.05 (GPT-3.5), and 0.87 ± 0.05~0.88 ± 0.05 (GPT-4.0). This study demonstrates the use of LLM model-extracted clinical information, in conjunction with imaging analysis, to improve the prediction of clinical outcomes, with bladder cancer as an initial example.

## 1. Introduction

Image interpretation in radiology is not a stand-alone task, but rather occurs in the context of available clinical information in the medical record, which must be manually extracted and mentally synthesized into a working pre-test context by both the ordering physician and the radiologist. Effectively, an ordering physician qualitatively or quantitatively assesses the probability of an outcome or disease state based on clinical data and determines the need for an imaging study. The radiologist interprets imaging data also in the context of this clinical information. The radiologist’s and the clinician’s dedication and ability to perform these tasks affect the appropriateness of exam selection, and quality of interpretation. LLMs could aid in these tasks to provide a more efficient interpretation, improved diagnosis and survival prediction, and more uniform patient care. Here, we explore the use of LLMs for extracting relevant clinical information and using this information to improve automated image analysis, using the prediction of bladder cancer survival as an initial model. 

Bladder cancer is the 6th most common cancer in the US, and the 4th most common cancer in men [1]. Expected survival duration is an important factor in optimizing patient management. Nomogram models using clinicopathological information can be used to predict survival in bladder cancer patients. The addition of imaging information can further improve the survival prediction accuracy of the models [2]. 

Numerous studies have shown that survival of bladder cancer patients may be predicted using clinicopathological information [3], histology [4], genetics [5], and molecular markers [6]. Analytic methods included risk stratification [7], visualized nomograms, and machine learning models such as support vector machines [8]. Nomogram models, which utilize clinicopathological information and are often based on large patient cohorts, can offer good accuracy in predicting survival. Multiple nomogram models have been proposed for bladder cancer, with concordance indices ranging from 63–66% [9,10] and AUC of 0.77–0.79 [11,12]. 

However, manual extraction of clinicopathological information from medical records is labor intensive and time consuming. An automated approach would be very useful. The rapid development of LLMs provides an opportunity to explore the use of LLMs for the automated extraction of information from clinical reports, which can be used as input to both nomogram models and integrated models, combining clinical and image information for survival prediction. 

LLMs have the potential to help with various aspects of patient care, including patient inquiries, note writing, decision-making, trial enrollment, data management, and patient education [13]. LLMs are not without problems, however. These include limitations such as hallucinations, factual inaccuracies, and bias [13]. In the context of diagnostic studies, there are specific challenges associated with disease diagnosis and the generation of comprehensive reports. The role of LLMs in diagnosis is being actively explored [14,15], with early results showing that these models can outperform laypeople, but fall short of matching the diagnostic capabilities of physicians. Despite these limitations, the careful incorporation of LLMs into diagnostic pathways could provide a useful aid to physicians. 

Imaging plays a key role in most diagnostic pathways. For bladder cancer patients, the CT urogram (CTU) is commonly utilized to provide important staging information for cancerous lesions. Using advanced image analysis approaches, such as radiomics and deep learning, has the potential to provide useful quantitative information regarding survival prediction [2].

We hypothesize that LLMs can automatically and accurately extract relevant clinicopathological information from medical records, and that integrated models based on both clinical and imaging information are more accurate in predicting bladder cancer patient survival than either channel alone. 

Developing a multi-modal predictive model for patient diagnosis and treatment decision-making can be achieved by integrating diverse types of data, such as clinical, genomic, imaging, and pathological data [16,17]. Such models offer several potential advantages. First, by incorporating various types of data, multi-modal models can capture more comprehensive and nuanced information about the patient’s condition, leading to enhanced predictive accuracy [18,19]. Second, they can provide a more holistic view of a patient’s health status, which is crucial for understanding complex diseases like cancer [20]. Third, multi-modal models are generally more robust regarding missing or noisy data, thereby creating models that are more generalizable across different patient cohorts and clinical settings [21]. Additionally, these models can help uncover the complex interactions and mechanisms underlying cancer progression and patient survival, contributing to the development of new therapeutic targets [22]. Due to limitations in data and resources, this study focuses specifically on integrating clinical and imaging data.

Here, we evaluate the capability of LLMs to extract key clinical information from medical records for input into a validated nomogram [11] to estimate bladder cancer patient survival, and we subsequently evaluate the use of this information in combination with imaging analysis for predicting bladder cancer patient survival after radical cystectomy. We investigated the use of multi-modal predictive models that integrate the LLM-aided clinical model (C) with radiomics (R) and/or deep learning (D) descriptors extracted from CTU images.

The expected direct contributions of this study include the following: (1) Demonstrating the use of LLMs in accelerating information retrieval from unstructured medical records. (2) Proposing novel LLM-aided multimodal predictive models (combining clinical and imaging data), with improved efficacy and efficiency in survival prediction for bladder cancer patients compared to our previous work [2]. 

## 2. Materials and Methods

### 2.1. Patient Cohorts

This retrospective study is HIPAA compliant and Institutional Review Board (IRB) approved. We collected clinical reports and CTU scans from 781 bladder cancer patients spanning 2001 to 2020 at one institution. 

To enhance the effectiveness of survival prediction models based on imaging data, we utilized pairs of pre- and post-chemotherapy CTU scans to delineate changes in bladder cancer following treatment. Eligible patients met these criteria: (1) having at least one set of pre- and post-chemotherapy CTU scans following neoadjuvant chemotherapy; (2) having undergone radical cystectomy; and (3) having available five-year follow-up data post-cystectomy to determine survival status. The reference standard for survival classification was determined by a research assistant (J.G.) using the patients’ follow-up data.

**Figure 1:**
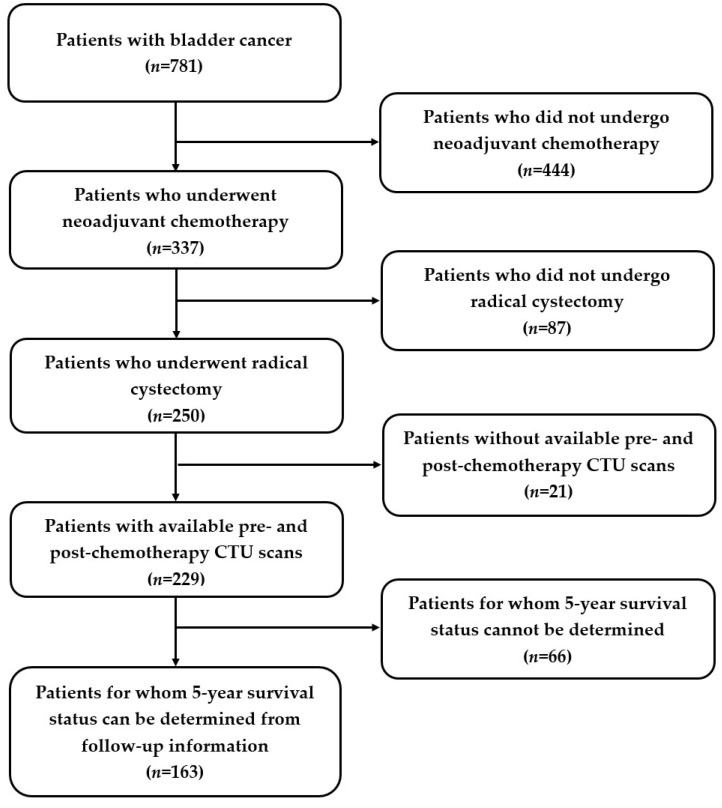
Study cohort diagram. Out of 781 patients, 163 patients (mean age 64 ± 9 years; M:F 131:32) were identified for this study. Patients who (1) underwent neoadjuvant chemotherapy and had pre- and post-chemotherapy CTU scans, (2) underwent radical cystectomy, and (3) had follow-up data to determine their five-year survival status were eligible.

Of the total of 781 bladder cancer patients, 163 patients satisfied the above criteria (Figure 1). Among these 163 patients, 48.5% (79/163) were alive five years after radical cystectomy, while 51.5% (84/163) died before reaching their fifth year post-cystectomy. We split the dataset into training, validation, and test sets: 56% (92/163) training (55 alive, 37 deceased); 4% (7/163) validation (4 alive, 3 deceased); and 40% (64/163) test (20 alive, 44 deceased), keeping a relatively large portion of the patients in both the training and test sets. The bladder cancer cases were collected chronologically, with medical records and exam dates in the training and validation sets dating from 2003 to 2016, and in the test set, dating from 2012 to 2020. This serial approach simulated, to some extent, the “real world” clinical situation, where the model is built based on previous cases and all weights and parameters are frozen after training, with the model then deployed to the new cases.

### 2.2. Predictive Models

**Figure 2:**
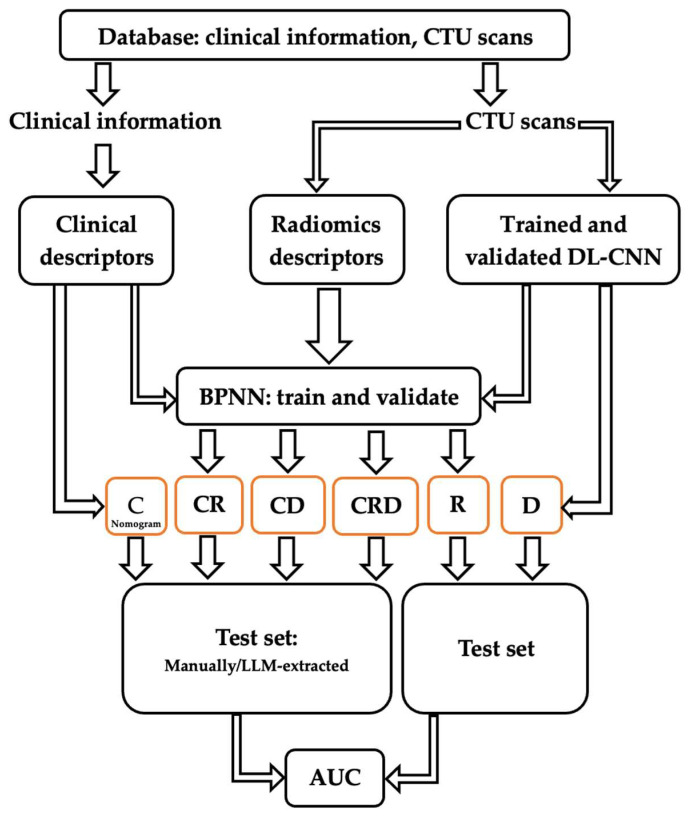
Development and validation processes of predictive models C, R, D, CR, CD, and CRD, using clinical and radiomics descriptors as well as a deep learning assessment.

The development process of the predictive models is illustrated in Figure 2. Comprehensive data were gathered, comprising clinical information and CTU scans. Clinical descriptors (C) relevant to the nomogram were extracted from clinical data, both manually and by LLMs, and used to calculate the survival probability [11]. Radiomics (R) descriptors were extracted from the CTU scans. Deep learning (D) estimation of patient survival was also obtained using CTU scans. Subsequently, individual and combined models based on the extracted descriptors were constructed. These models were then deployed on an independent test set to evaluate their performance in predicting survival.

#### 2.2.1. Clinical (C) Descriptors

Five clinical descriptors are needed for input into a commonly used nomogram [11]—(1) post-surgery pathologic T stage, (2) pathologic node (N) stage, (3) presence or absence of lymphovascular invasion (LVI), (4) whether patients underwent neoadjuvant chemotherapy, and (5) whether patients underwent adjuvant radiotherapy. In this study, the last two descriptors remained constant; that is, all 163 patients underwent neoadjuvant chemotherapy, and none underwent adjuvant radiotherapy. Thus, only the three remaining extracted descriptors were variable: (1) post-surgery pathologic T stage, (2) pathologic N stage, and (3) presence or absence of LVI. 

The three clinical descriptors (C descriptors) were extracted manually from clinical reports by the research assistant (J.G.) and reviewed by a radiologist with 37 years of experience (R.H.C.). They confirmed that each patient’s record included relevant information about the pathologic T stage, N stage, and LVI. The manually extracted C descriptors were used as the reference standard. 

The C descriptors were also extracted using LLMs. We employed five different advanced LLMs, including Dolly-v2 [23], Vicuna-v1.3-13b [24], Llama-2.0-13b [25], GPT-3.5 [26], and GPT-4.0 [27]. 

We fine-tuned the key parameters of Dolly, Vicuna, and Llama to optimize their performances. These parameters were finalized through deploying the LLMs to cases from the training set (temperature = 0.2; repetition penalty = 0.0). We ran GPT-3.5 and GPT-4.0 directly without fine-tuning their parameters.

LLM prompts consisted of three parts: (1) task description, (2) current input, and (3) output indicator. To evaluate the accuracy and robustness of LLMs in extracting information from medical reports, we designed two prompts with different task descriptions and output indicators by using a sample of medical reports and modifying the prompts until the LLM output was relatively stable and accurate. We used Dolly as the LLM for this task. The first prompt had a relatively general request, while the second prompt had a more specific request with constraints. We assigned one user to apply the first prompt (User1 prompt), and the other user to apply the second prompt (User2 prompt):

- **User1 prompt:** “Please find the pathologic stage (revealed by cystectomy or in the assessment section), Pathologic node stage, lymphovascular invasion (LVI), Angiolymphatic invasion”.
- **User2 prompt:** “Using only the following information, find the pathologic stage (revealed by cystectomy or in the assessment section), node stage, angiolymphatic or lymphovascular invasion (LVI), and write the answers in the format of a list. If the answers are not in the information, write “unspecified””.

LLMs have limitations on input text length (2048 tokens for Dolly-v2, 2048 tokens for Vicuna-13b, 4096 tokens for Llama-2.0-13b, 4096 tokens for GPT-3.5, and 8192 tokens for GPT-4.0). Typically, the three descriptors needed were included in the pathological report from the radical cystectomy. However, the full length of the surgery report often surpassed the word limit for Dolly and Vicuna. To address this, the reports were partitioned by the research assistant by selecting the portion of the report containing the relevant information. For some cases, the three descriptors were found in different reports or in different sections of a long report. In these instances, we manually consolidated the parts containing relevant information from multiple reports or from different sections. This simulated a single report within the maximum length that can be analyzed by all five LLMs being compared. To minimize the effect of the manual manipulation, we strictly adhered to the following rules: other than the necessary combination operation, no additional editing was done—such as removing sentences, changing sentence sequence, or modifying the format and layout—to preserve the original writing of the clinical reports. We named both partitioned (*n* = 130) and consolidated (*n* = 33) reports as “snipped reports”. To evaluate the relative performances of the LLMs, we fed the same set of snippet reports into the five LLMs. Additionally, we conducted supplementary experiments by inputting the full reports into GPT-3.5 and GPT-4.0. In the event that the three descriptors were found in different reports, these full reports were concatenated. We named both the full reports (*n* = 130) and the concatenated full reports (*n* = 33) as “full reports”. This aimed to assess the impact of input length on the information extraction ability of the LLMs.

#### 2.2.2. Radiomics (R) Descriptors and Deep Learning (D) Assessment

**Figure 3:**
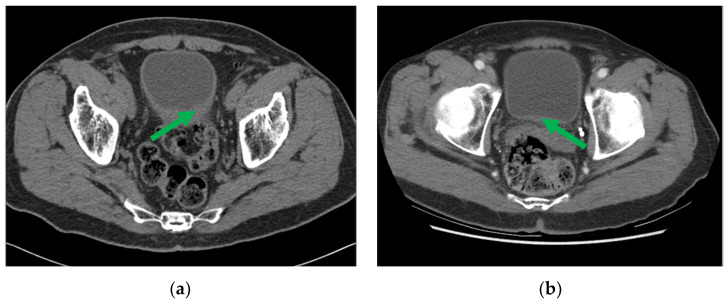
CTU images of a bladder cancer patient (**a**) pre- and (**b**) post-neoadjuvant chemotherapy, with the initial and residual cancer indicated by the green arrow.

One example of pre- and post-chemotherapy CTU scans of bladder cancer can be found in Figure 3. The CTU scans used in this study were acquired with GE Healthcare LightSpeed MDCT scanners, Issaquah, WA, USA, set at 120 kVp and 120–280 mA, with the slice interval ranging from 0.625 to 5 mm. The CTU series with the comparatively best quality were utilized regardless of whether they were performed after administration of contrast material or were non-contrast images. The contrast-enhanced CTU scans were obtained 12 min after the initiation of the first bolus of a split-bolus IV contrast injection, and 2 min after the initiation of the second bolus of 175 mL of nonionic contrast material at a concentration of 300 mg iodine/ml. 

The CTU scans were interpreted by the radiologist (R.H.C.), who marked the volume of interest (VOIs) and the slice where the lesion had the most prominent appearance. The lesion was then automatically segmented with our in-house-developed Auto-Initialized Cascaded Level Set (AI-CALS) model [28] within the VOI. 

##### Radiomics (R) Descriptors:

A set of 91 radiomics features (RF), including morphological, texture, and intensity features, were extracted from the segmented lesions. This set of features has demonstrated excellent performance in various tasks, such as lung nodule classification [29] and bladder cancer treatment response assessment [30]. We denoted RF extracted from pre- and post-chemotherapy treatment scans as $f_{pre}$ and $f_{post}$, respectively. To capture the changes in lesion features between the pre- and post-treatment scans, we calculated difference features $f_{diff}$ as follows:

$$f_{diff}=\frac{f_{pre}-f_{post}}{f_{pre}}.$$

This resulted in a total of 273 RF ($f_{pre},\;f_{post},\;f_{diff}$). It is crucial to eliminate redundant or ineffective features, thereby reducing the time and space requirements for data processing, as well as the risk of the “curse of dimensionality” when the training data size is limited. We performed feature selection on training and validation sets, using Joint Mutual Information Maximization (JMIM) [31] alongside k-fold cross validation, which resulted in 12 features being selected as the R descriptors.

##### Deep Learning (D) Assessment:

To obtain deep learning survival likelihood scores from the lesions on the pre- and post-treatment CTU scans, we utilized the ImageNet pre-trained Inception v1 model, a 22-layer deep (27 layers including the pooling layers) neural network structure [32]. Within the segmented lesion area, we employed a sliding window technique to extract ROIs of 32 × 16 pixels in size. One ROI from the pre-chemotherapy scan and one from the post-chemotherapy scan were combined into a hybrid ROI (32 × 32 pixels). The hybrid ROIs were enlarged (by cubic spline interpolation) to 224 × 224 pixels, to align with the input specification of Inception v1. Each scan pair could provide a number of hybrid ROIs. To ensure a balanced dataset and prevent dominance or bias caused by CTU scan pairs from large lesions, we imposed a threshold to limit the number of hybrid ROIs from one scan pair by randomly eliminating excess hybrid ROIs. All hybrid ROIs from the same patient were assigned the same case label: survived for 5 or more years = 1; otherwise = 0. The Inception v1 model provided a likelihood score as an output for each input hybrid ROI. The likelihood scores of all hybrid ROIs from the same patient were combined into a single likelihood score reflecting the survival likelihood of that patient.

#### 2.2.3. Survival Prediction Models

Clinical descriptors were input into the nomogram for survival prediction [11], requiring no additional training since the nomogram was developed previously and can be applied directly to new cases. Radiomics descriptors were input to a backpropagation neural network (BPNN) [33] to generate a survival likelihood score. The Inception v1 model [32] utilized hybrid ROIs from pre- and post-chemotherapy CTU scans to generate a survival likelihood score. Both the parameters of the BPNN and the Inception v1 model were trained and optimized using the training and validation sets. As a result, we obtained three individual models predicting five-year survival: C (utilizing clinical descriptors), R (utilizing radiomics descriptors), and D (providing deep learning assessment). 

To integrate the image descriptors with the clinical descriptors, we developed three combined models—CR (combining the clinical and radiomics descriptors), CD (combining the clinical descriptors and the deep learning assessment), and CRD (combining the clinical and radiomics descriptors, and the deep learning assessment)—by using the information from these two or three sources as input into a BPNN for survival prediction. The parameters of this BPNN were also optimized using the training and validation sets. 

#### 2.2.4. LLM Direct Survival Prediction

We also evaluated the ability of GPT-4.0 to directly predict the five-year survival of bladder cancer patients based solely on raw clinical reports. We selected GPT-4.0 because it was the most advanced LLM available to us. We crafted a prompt (SP prompt) to ask GPT-4.0 to predict survival probabilities and cite sources that supported its prediction:

- **SP prompt:** “Using only the following information, determine the patient’s 5-year bladder cancer survival prediction (in the format of a probability) based on the pathologic stage (revealed by cystectomy or in the assessment section), node stage, and angiolymphatic or lymphovascular invasion (LVI). You must provide a quantitative probability even if you’re unable to make an exact estimation. Provide the references and sources used to make the survival prediction. This is not to be considered as medical advice”.

We designed the SP prompt by using a sample of medical reports and modifying the prompts until the LLM output was relevant and relatively stable.

### 2.3. Statistical Analysis

The LLM–extracted clinical information was compared with manually extracted information (reference standard). Extraction accuracy was evaluated based on the degree of agreement between the clinical information extracted by LLMs and that extracted manually. Each predictive model’s performance was evaluated using the area under the receiver operating characteristic (ROC) curve (AUC), calculated using the LABROC program [34]. The statistical significance of the differences in model performance was determined using iMRMC software (version 4.03; U.S. Food and Drug Administration; https://github.com/DIDSR/iMRMC/releases, accessed on 15 December 2023) [35], with the critical alpha value adjusted for multiple hypothesis testing using the Bonferroni correction [36]. Kaplan–Meier analyses [37] and log-rank tests were conducted to generate survival curves. Patients in the test set were stratified into predicted deceased and alive groups using the decision thresholds on the BPNN output scores. These thresholds were determined based on the minimal misclassification points for the C and CRD models, separately derived from the training and validation sets. Additionally, to evaluate GPT-4.0, the percentage of hallucinations was calculated, which determined instances where irrelevant sources were cited for survival predictions.

## 3. Results

### 3.1. Patient Characteristics

From 781 bladder cancer patients, 163 (mean age 64 ± 9 years, M:F 131:32) formed the study cohort. Table 1 shows their demographics and key clinical information, including gender, age at surgery, tobacco use, post-surgery pathologic stage, pathologic node stage, LVI, and whether patients underwent neoadjuvant chemotherapy or adjuvant radiotherapy.

### 3.2. LLM Accuracy in Extracting Information

The accuracy of the five LLMs in extracting the three clinical descriptors of 163 patients is presented in Table 2. GPT-4.0-snippet-report demonstrated the highest average accuracy for both users, achieving 97% (User1) and 96% (User2). The average accuracy of GPT-4.0-full-report was 94% (User1) and 95% (User2). Dolly, Vicuna, Llama, and GPT-3.5 exhibited average accuracies ranging from 74% to 93%. Table 2 also shows the corresponding AUCs for survival prediction of each of the C models. Note that, for a given LLM, a positive correlation is observed between higher average accuracy and increased survival AUC. The differences between the predictions by the C models based on the manually extracted and the LLM-extracted clinical descriptors did not reach statistical significance.

### 3.3. Five-Year Survival Prediction

Table 3 presents the performances of the survival predictive models, C, CR, CD, and CRD, alongside the accuracy of the LLMs (average over three descriptors or only pathologic T stage) on the test set (*n* = 64). For the survival predictions by the models (C or CRD), the difference between using the manually extracted (reference standard) and the LLM-extracted (with either the User1 or User2 prompt) clinical descriptors as input to the nomogram did not reach statistical significance. 

**Figure 4:**
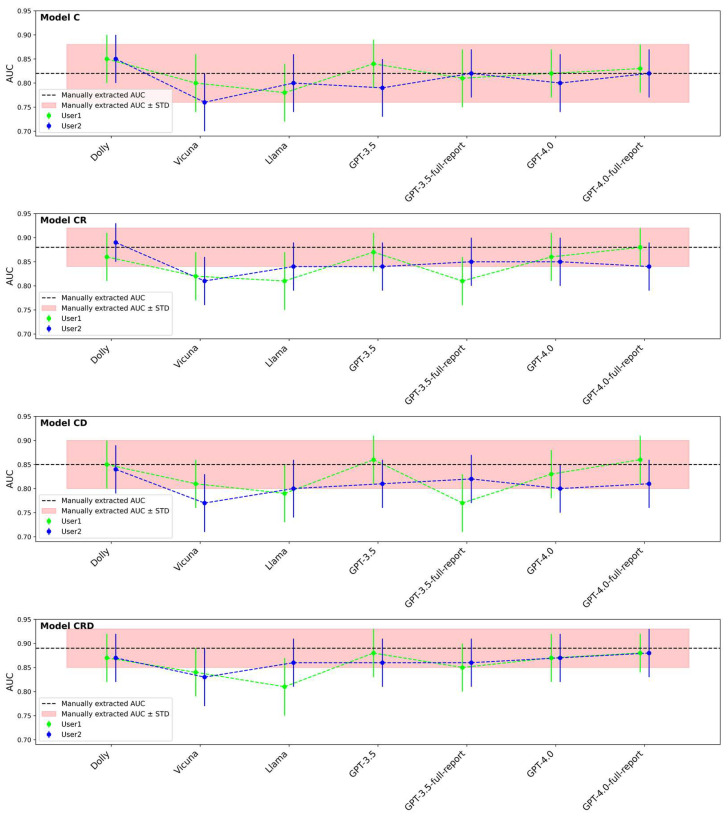
AUCs of LLM-aided models (C, CR, CD, CRD) compared to AUCs based on manually extracted clinical descriptors. Clinical information was extracted manually (reference standard) and by LLMs with User1 prompt or User2 prompt.

**Figure 5:**
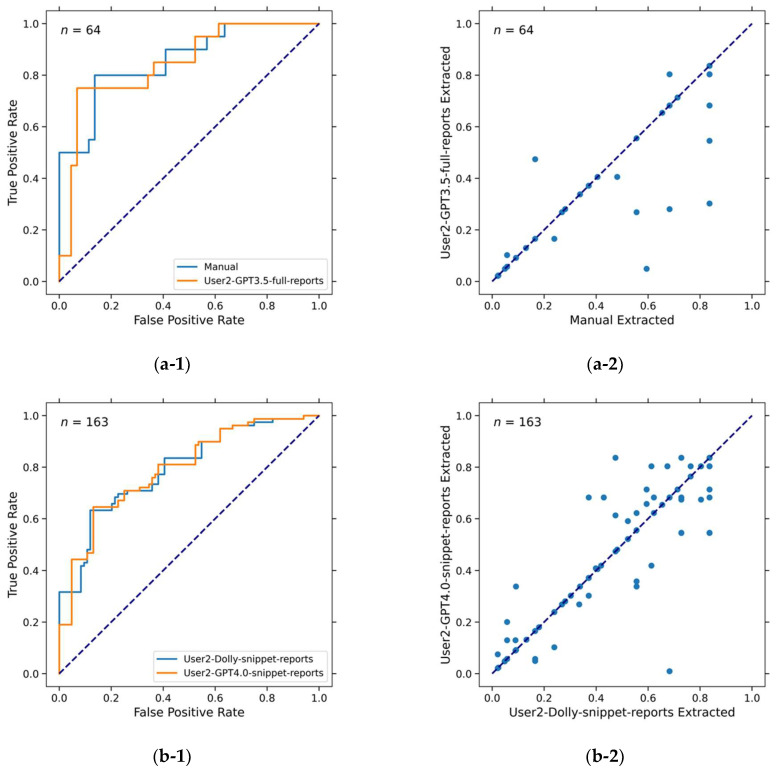
ROC curves and scatter plots showing that different models have the same AUC values but different predictions (survival likelihood scores). (**a-1**) ROC curves of predictions by model C-manual and model C-User2-GPT3.5-full-reports. Both had AUC = 0.82 ± 0.06. (**a-2**) Scatter plot of predictions (survival likelihood) by model C-manual and model C-User2-GPT3.5-full-reports. Pearson correlation coefficient is 0.90. (**b-1**) ROC curves of predictions by model C-User2-Dolly-snippet-report (accuracy = 87%, AUC = 0.77 ± 0.04) and model C-User2-GPT4.0-snippet-reports (accuracy = 96%, AUC = 0.77 ± 0.04). (**b-2**) Scatter plot of predictions (survival likelihood) by model C-User2-Dolly-snippet-report and model C-User2-GPT4.0-snippet-reports. Pearson correlation coefficient is 0.95.

Figure 4 compares the AUCs of the C, CR, CD, and CRD survival models based on the manually extracted and LLM-extracted descriptors. To be of any clinical utility, the target performance for models based on LLM-extracted clinical descriptors should be at least the same as that of the models based on manually extracted (reference standard) clinical descriptors. The deviation of LLM-aided models from the reference standard models would be a result of inaccurate clinical descriptor extraction by LLMs. It is worth mentioning that even if two models have identical AUCs, the survival likelihood scores of the cases may still be different. Two examples that had the same AUCs but different scores are shown in Figure 5. 

The R, D, and RD models achieved relatively low performances, with AUCs of 0.74 ± 0.07, 0.69 ± 0.08, and 0.76 ± 0.07, respectively. However, when they were combined with the C model, the overall performance improved substantially, as shown in Table 3. Before the Bonferroni correction, C-manual vs CRD-manual, R vs CRD-manual, and D vs CRD-manual all achieved statistical significance (*p* = 0.019, *p* = 0.019, *p* = 0.009, respectively). 

**Figure 6:**
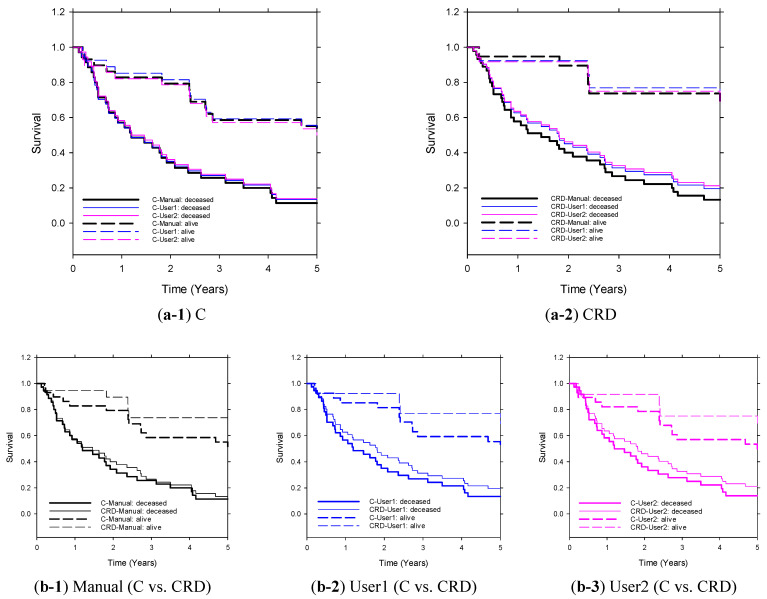
Kaplan–Meier survival curves of C and CRD models (User1/User2-GPT4.0-snippet-reports) on the independent test set (*n* = 64). The clinical descriptors were extracted manually (reference standard) or by LLM through User1 prompt or User2 prompt. Stratification of the two groups (predicted deceased and predicted alive) by C and CRD (extracted by manual or GPT4.0-snippet-reports) achieved statistical significance (*p* ≤ 0.05).

The Kaplan–Meier analysis shown in Figure 6 demonstrates that the CRD model has outperformed the C model in five-year survival prediction. Once again, the models based on LLM-extracted descriptors exhibited comparable performances to the models based on manually extracted descriptors.

### 3.4. LLM Direct Survival Prediction

GPT-4.0 provided survival probability and cited sources that supported its prediction using the information from each patient’s clinical report. For the direct prediction of five-year survival by LLMs (Table 4) on the entire set of 163 patients, GPT-4.0 attained an AUC of 0.76 ± 0.04 and 0.73 ± 0.04 for the snippet report and full report input, respectively; for the subset of 64 patients, GPT-4.0 attained an AUC of 0.81 ± 0.05 and 0.75 ± 0.07 for the snippet report and full report input, respectively. Note that for this experiment, both sets were independent test sets. Regarding the sources that GPT-4.0 provided as the references from which it obtained the predictions for the entire set of 163 patients, GPT-4.0 had a hallucination rate of 32.0% and 17.2% for the snippet report and full report input, respectively; for the subset of 64 patients, GPT-4.0 had a hallucination rate of 37.5% and 12.5% for the snippet report and full report input, respectively.

## 4. Discussion

This study introduced a viable approach that incorporates large language model medical record extraction with radiomic and deep learning analysis of imaging studies in bladder cancer patients. The combination of LLM-obtained clinical information and machine learning image analysis enhanced the accuracy of predicting five-year survival for bladder cancer patients after radical cystectomy, achieving an AUC of 0.88 ± 0.05, as compared to an AUC of 0.82 ± 0.06 achieved by manually extracted clinical information alone; an AUC of 0.82 ± 0.05 achieved by LLM-extracted clinical information alone; and a range of 0.69 ± 0.08~0.76 ± 0.07 achieved by computational analysis of imaging information alone. The differences between the individual combined models are not statistically significant. 

From two comprehensive reviews [38,39] on bladder cancer outcome prediction, frequently used techniques for survival prediction have included nomograms, artificial neural networks, and risk stratification. The bladder cancer-specific survival nomogram from Shariat’s well-cited work is a reliable model based on clinical information, achieving an AUC of 0.79 [11]. In this study, by capitalizing on the advantages of a nomogram, as well as harnessing the power of deep learning in extracting image information and the strengths of radiomics analysis, we integrated multi-modal data sources into an effective combined model for survival prediction. 

The C model is based on a widely accepted nomogram that exhibits a commendable predictive performance regardless of whether the input clinical information is extracted manually or by LLMs. The assistance of LLMs could certainly streamline the use of nomograms by reducing the workload involved in extracting key information from extensive report reviews. In this context, we exploited the information summarization and extraction capabilities of LLMs. Table 3 shows that a more detailed prompt with proper constraints improves the average accuracy of LLM information extraction and, thus, likely provides more reliable survival predictions. 

The combined models, utilizing CT urogram (CTU) image analysis in addition to the C model, achieved higher performances than clinical model C alone, with either the manually or LLM-extracted information. This finding demonstrates that combined clinical and imaging models have the potential to improve prediction of patient survival. 

For GPT-3.5 with the general prompt (User1 Prompt), the full report input led to a lower accuracy of clinical information extraction and a larger AUC deviation from the reference standard-derived AUC, while using a more constrained prompt (User2 Prompt) meant that the impact of input report length on the accuracy and the AUC was less evident. Interestingly, for GPT-4.0, input report length had essentially no effect on AUC, regardless of using a general or constrained prompt. 

The direct prediction performance of five-year patient survival by GPT-4.0 was slightly lower than those of the other C or CRD models. This is likely explained by the fact that GPT-4.0 had a hallucination rate of about 35% when the inputs were snippet reports, to only about 15% when the inputs were full reports. These results suggest that more information from the medical reports might help GPT-4.0 find the online resources more accurately. 

This study has several important limitations. First, the overall training dataset was of limited size. However, it should be pointed out that the R, D, and combined models were pre-trained, while the nomogram was developed from a larger independent patient cohort [11]. Although the models utilizing image descriptors (R and D models) were not optimally trained due to the small training set size, this study demonstrates the promise of the proposed combined LLM and imaging analysis approach to survival prediction. 

Second, while the overall performance of CRD was improved substantially compared to the C, R, and D models (achieving statistical significance for manually extracted clinical information (*p* < 0.02)), the comparisons between the predictive models did not achieve statistical significance after the Bonferroni correction was made. Larger datasets may reveal statistical differences between the combined models. 

Third, LLMs are prone to hallucinations, although it is expected that more domain-specific training may alleviate the problem. 

Fourth, it may be more informative if more different user prompts are used to further evaluate the accuracy and the reproducibility of information extraction by the LLMs. 

Finally, the extraction of information to be used as input to the nomogram was performed from curated off-line data. For optimal clinical impact, this process will have to become fully automated, with the data extracted directly from electronic medical records in the absence of any curation. 

The results have important clinical implications. If LLMs could automatically estimate the pre-test probability of survival (or any other desired clinical outcome), the data could provide important clinical context for ordering studies. Exams that likely would not provide additive diagnostic utility based on pre-test analysis could be avoided, with implications for maximizing resource management, reducing patient stress, and decreasing burdens on a stretched and chronically short-staffed workforce. Further, the radiologist can use this information as a basis for image interpretation, improving the interpretive process by adding objectivity and uniformity to pre-test information gathering and clinical decision-making. Finally, this work offers the possibility of improving radiomics- and machine learning-based interpretive tools of imaging studies by adding clinical information to the analysis. 

Some directions for further improving this study include the following: (1) Enhancing information retrieval accuracy by employing more comprehensive prompt engineering for LLMs. (2) Increasing the volume of data collection and exploring novel deep learning models to extract more nuanced imaging features from CT scans. (3) Improving descriptor/feature fusion techniques. While this study utilized a late fusion technique, future works could investigate early fusion (such as feature concatenation), attention mechanisms, multi-scale fusion, or decision-level fusion.

## 5. Conclusions

This study shows that LLMs can extract clinical information accurately from a patient’s medical records and a patient’s medical reports. Models utilizing LLM-extracted clinical descriptors as input to a nomogram predicting 5-year post-cystectomy bladder cancer survival achieve comparable performances to those of models utilizing manually extracted clinical descriptors. The study also demonstrates the efficacy of combining automatically extracted clinical and imaging features to improve the performance of survival predictive models for bladder cancer patients.

## Figures and Tables

**Table 1:** Demographics and key clinical information (*n* = 163) of the patient cohort.

Attributes		# of Patients
Gender	Male	131
	Female	32
Age at surgery	Mean age ± standard deviation	64 ± 9
Tobacco use	Current	40
	Former	88
	Never	34
	Unknown	1
Pathologic T stage	pT0	35
	pTa/pTi/pTis	15
	pT1	16
	pT2	36
	pT3	45
	pT4	16
Pathologic N stage	N0	112
	N1	24
	N2	23
	N3	4
LVI ^1^	Yes	61
	No	102
Neoadjuvant chemotherapy	Yes	163
	No	0
Adjuvant radiotherapy	Yes	0
	No	163

^1^ LVI = lymphovascular invasion.

**Table 2:** The average performances of the LLMs in clinical descriptor extraction with User1 prompt and User2 prompt, and the survival prediction performances of the C models (*n* = 163). The entire set was a test set because none of the cases were used for training the LLMs or the C models.

*n* = 163								
Ground Truth	Prompt	Dolly	Vicuna	Llama	GPT-3.5	GPT-3.5-full-report	GPT-4.0	GPT-4.0-full-report
	LLM ^1^ accuracy							
100%	User1	74%	76%	82%	87%	85%	97%	94%
	User2	87%	83%	93%	91%	91%	96%	95%
	AUC ^2^ ± standard deviation (C model ^3^)							
0.78 ± 0.04	User1	0.74 ± 0.04	0.70 ± 0.04	0.73 ± 0.04	0.76 ± 0.04	0.74 ± 0.04	0.77 ± 0.04	0.77 ± 0.04
	User2	0.77 ± 0.04	0.72 ± 0.04	0.75 ± 0.04	0.75 ± 0.04	0.77 ± 0.04	0.77 ± 0.04	0.77 ± 0.04

^1^ LLM = large language model; ^2^ AUC = area under the receiver operating characteristic curve; ^3^ C model = model based on clinical descriptors.

**Table 3:** Performances of survival predictive models C, CR, CD, CRD on the independent test set (*n* = 64), measured in AUC, alongside the accuracy of LLMs in extracting clinical descriptors. Clinical information was extracted manually (reference standard) and by LLMs with User1 prompt or User2 prompt.

Test set (*n* = 64)									
	Manual	Prompt	Dolly	Vicuna	Llama	GPT-3.5	GPT-3.5-full-report	GPT-4.0	GPT-4.0-full-report
			LLM ^1^ accuracy						
Average accuracy of pathologic T stage, N stage, and LVI ^2^	-	User1	85%	80%	88%	92%	87%	95%	95%
	-	User2	93%	87%	93%	94%	91%	94%	94%
Pathologic T stage	-	User1	92%	88%	89%	94%	88%	97%	97%
	-	User2	94%	84%	91%	88%	86%	88%	91%
			AUC ^3^ ± standard deviation						
C ^4^	0.82 ± 0.06	User1	0.85 ± 0.05	0.80 ± 0.06	0.78 ± 0.06	0.84 ± 0.05	0.81 ± 0.06	0.82 ± 0.05	0.83 ± 0.05
		User2	0.85 ± 0.05	0.76 ± 0.06	0.80 ± 0.06	0.79 ± 0.06	0.82 ± 0.05	0.80 ± 0.06	0.82 ± 0.05
CR ^5^	0.88 ± 0.04	User1	0.86 ± 0.05	0.82 ± 0.05	0.81 ± 0.06	0.87 ± 0.04	0.81 ± 0.05	0.86 ± 0.05	0.88 ± 0.04
		User2	0.89 ± 0.04	0.81 ± 0.05	0.84 ± 0.05	0.84 ± 0.05	0.85 ± 0.05	0.85 ± 0.05	0.84 ± 0.05
CD ^6^	0.85 ± 0.05	User1	0.85 ± 0.05	0.81 ± 0.05	0.79 ± 0.06	0.86 ± 0.05	0.77 ± 0.06	0.83 ± 0.05	0.86 ± 0.05
		User2	0.84 ± 0.05	0.77 ± 0.06	0.80 ± 0.06	0.81 ± 0.05	0.82 ± 0.05	0.80 ± 0.05	0.81 ± 0.05
CRD ^7^	0.89 ± 0.04	User1	0.87 ± 0.05	0.84 ± 0.05	0.81 ± 0.06	0.88 ± 0.05	0.85 ± 0.05	0.87 ± 0.05	0.88 ± 0.04
		User2	0.87 ± 0.05	0.83 ± 0.06	0.86 ± 0.05	0.86 ± 0.05	0.86 ± 0.05	0.87 ± 0.05	0.88 ± 0.05

^1^ LLM = large language model; ^2^ LVI = lymphovascular invasion; ^3^ AUC = area under the receiver operating characteristic curve; ^4^ C model = model based on clinical descriptors; ^5^ CR = model based on clinical and radiomics descriptors; ^6^ CD = model based on clinical descriptors and deep learning assessment; ^7^ CRD = model based on clinical and radiomics descriptors, as well as deep learning assessment.

**Table 4:** The direct survival predictions by GPT-4.0 with the SP prompt (survival prediction prompt). AUCs were calculated for the predictions of test sets of 163 patients and 64 patients. The LLM-cited sources, along with the predictions, were examined. Irrelevant sources cited by the LLM were defined as hallucinations.

	*n* = 163		*n* = 64	
	Snippet report	Full report	Snippet report	Full report
AUC ^1^ ± standard deviation	0.76 ± 0.04	0.73 ± 0.04	0.81 ± 0.05	0.75 ± 0.07
Source relevant	68.0% (111/163)	82.8% (135/163)	62.5% (40/64)	87.5% (56/64)
Source irrelevant (hallucination)	32.0% (52/163)	17.2% (28/163)	37.5% (24/64)	12.5% (8/64)

^1^ AUC = area under the receiver operating characteristic curve.

## Data Availability

Data are available upon request.
